# Recent Advances in the Use of the Dimerization Strategy as a Means to Increase the Biological Potential of Natural or Synthetic Molecules

**DOI:** 10.3390/molecules26082340

**Published:** 2021-04-17

**Authors:** Alexis Paquin, Carlos Reyes-Moreno, Gervais Bérubé

**Affiliations:** 1 Department of Chemistry-Biochemistry and Physics, University of Québec at Trois-Rivières, C.P. 500, Trois-Rivières, QC G9A 5H7, Canada; Alexis.Paquin@uqtr.ca; 2 Groupe de Recherche en Signalisation Cellulaire, University of Québec at Trois-Rivières, C.P. 500, Trois-Rivières, QC G9A 5H7, Canada; Carlos.Reyes-Moreno@uqtr.ca; 3 Department of Medical Biology, University of Québec at Trois-Rivières, C.P. 500, Trois-Rivières, QC G9A 5H7, Canada

**Keywords:** antitumor agents, biological activity, *C*_2_-symmetry, dimers, drug design, synthesis

## Abstract

The design of *C*_2_-symmetric biologically active molecules is a subject of interest to the scientific community. It provides the possibility of discovering medicine with higher biological potential than the parent drugs. Such molecules are generally produced by classic chemistry, considering the shortness of reaction sequence and the efficacy for each step. This review describes and analyzes recent advances in the field and emphasizes selected *C*_2_-symmetric molecules (or axial symmetric molecules) made during the last 10 years. However, the description of the dimers is contextualized by prior work allowing its development, and they are categorized by their structure and/or by their properties. Hence, this review presents dimers composed of steroids, sugars, and nucleosides; known and synthetic anticancer agents; polyphenol compounds; terpenes, known and synthetic antibacterial agents; and natural products. A special focus on the anticancer potential of the dimers transpires throughout the review, notwithstanding their structure and/or primary biological properties.

## 1. Introduction

**Figure 1:**
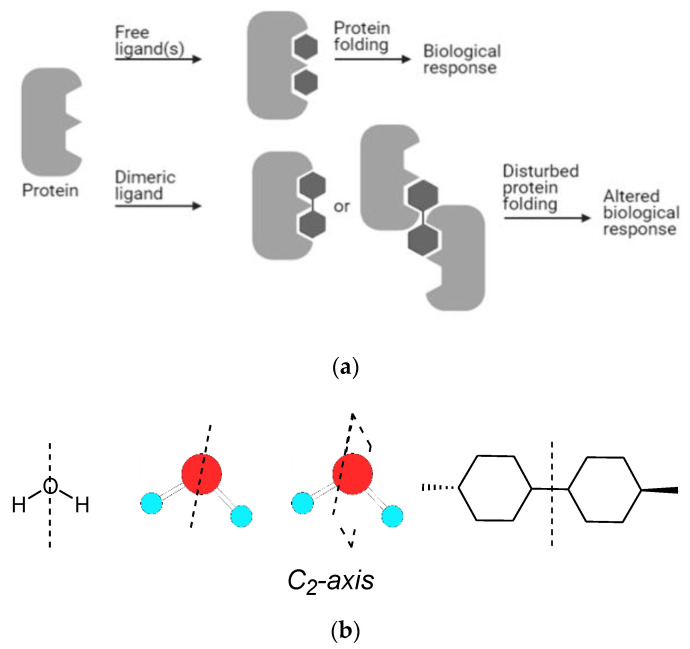
(**a**) Schematic representation of a dimeric molecule interacting with its receptor and biological outcome. (**b**) Schematic representations of *C*_2_-sigma plane or *C*_2_-axis symmetry using small molecules.

The synthesis of dimeric molecules has attracted considerable attention over the years. Dimers of biologically active molecules quite often show higher activity than the monomeric unit [1]. Many biological receptors or targets, once activated, dimerize upon an initial interaction with a drug. Thus, a dimer that could interact with such targets was imagined to be able to produce a stronger biological response than the parent drug. A dimeric drug could accommodate two independent binding sites on a receptor molecule, leading to a thermodynamically stronger interaction than that obtained by the attachment of two monomeric drugs (Figure 1a) [2,3]. Hence, this strategy was exploited for the construction of many types of drugs for the discovery of cutting-edge and innovative therapeutics.

The motivation for the design of dimers evolves from the fact that in natural products, molecular bilateral symmetry is found in about 7% of all isolated molecules, which represents a higher number than that estimated on coincidence [4]. Particularly, the *C*_2_-axis represents 69% of the total number of naturally occurring dimers. Generally, the biosynthesis of dimeric natural molecules occurs by a head-on approach of two identical units. So once again, Mother Nature inspires researchers to construct symmetrical therapeutic molecules [4]. Figure 1b displays this particular type of *C*_2_ symmetry (sigma plane or axis) that is often utilized by researchers to construct dimeric molecules. This topic was the subject of several reviews in the field of anticancer drugs [5], in the field of steroids [6], and recently as bioactive oligovalent symmetrical molecules [7].

Herein, we describe the most recent advances in this rich and vast research domain with a focus on the last decade (2010–2020). This review is divided into important types of biologically active molecules and emphasis on the earlier work that allowed the development of the reported dimers. The selection of the topics was based on recently reported studies in the literature. This review is divided into the following sections: steroids dimers and non-steroidal analogues, sugars and nucleoside-based dimers, dimers of known and synthetic anticancer agents, polyphenol dimers, terpenoid dimers, dimers of known and synthetic antibacterial agents, and recently isolated dimeric natural products. The molecules are categorized by their respective types to contrast the original medicinal properties with that of the corresponding dimers. Furthermore, the different sections were also selected as the authors of the studies express the need for designing a dimer to improve the activity of the basic molecules. The selection of compounds was also guided by the relevance of the described biological data.

It is important to specify that dimeric drugs are mainly intended to link two receptors, inhibiting the usual response to a ligand and frequently synergizing this response. Some dimeric molecules can be made to alkylate DNA inhibiting cell growth. The design of dimers will be guided by the intended molecular target. Most often, dimeric drugs are not released (or cleaved) within the cellular component of the cell, so they can act as a completely new molecular unit inside the targeted cells. Furthermore, such dimers are not meant to follow Lipinski’s rule of five, which applies to small molecular entities.

## 2. Steroids Dimers and Non-Steroidal Analogs

**Figure 2:**
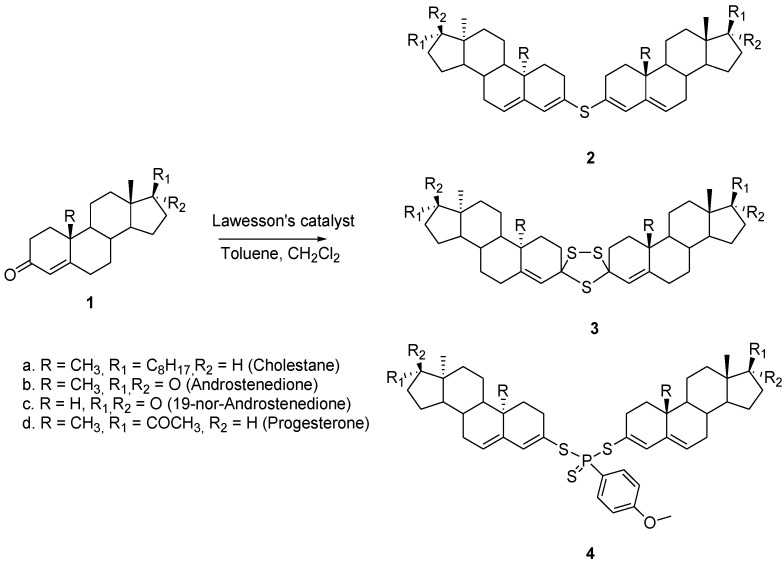
Synthesis of symmetric (**3**,**4**) and asymmetric (**4**) steroid dimers with a sulfur-based bridge.

Steroids play an important biological role in nature. Hence, the dimerization strategy was applied to steroids with the goal of improving their biological potential. Several dimers were fabricated by reaction of steroidal compounds (**1a**–**d**) with Lawesson’s catalyst [8]. According to the reaction conditions, different proportions of the dimers **2**, **3,** and **4** with distinct linkers were isolated and characterized (Figure 2). These dimers were tested for their biological activity and the results showed the dimer with a sulfur ether bridge, the bis(cholesta-3,5-dien-3-yl) sulfide **2**, was the most active compound [9]. The doses having antiproliferative activity of this particular sulfide on cervical cancer (HeLa), breast cancers (MDA-MB-453 and MDA-MB-361), and leukemia (K562) human cell lines ranged from 14.9 to 27.1 μM in comparison to cisplatin, ranging from 2.1 to 17.1 μM. The dimeric compounds showed no antimicrobial activity; however, those linked with trithiolane ring system **3** exhibited antifungal activity against *Saccharomyces cerevisiae*. Despite the fact that the dimers are interesting molecules, the synthesis is rather difficult as it leads to a complex mixture of compounds.

**Figure 3:**
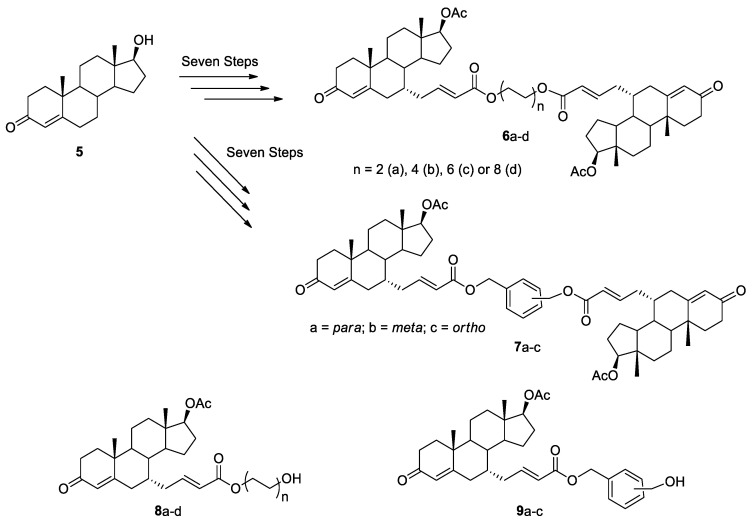
Synthesis of *C*_2_-symmetric testosterone dimers **6a**–**d** and **7a**–**c** by esterification reaction and their precursor’s **8a**–**d** and **9a**–**c**.

In another study, Vesper et al. reported the synthesis of novel *C*_2_-symmetric testosterone dimers were linked at position 7α of the steroid nucleus [10]. Two series of dimers were constructed from testosterone (**5**) using either a α,ω-aliphatic diol or an aromatic regioisomeric diol (*ortho*, *meta*, or *para*) as tether chains to produce dimers **6a**–**d** and **7a**–**c**, respectively (Figure 3). The dimers were constructed in a stepwise manner via the corresponding ester units **8a**–**d** and **9a**–**c**, allowing comparison of the antiproliferative activity with the final dimers. The most active dimer **6a** (n = 1) showed an IC_50_ of 3.8, 1.4, and 1.8 μM on LNCaP (AR+), DU-145 (AR−), and PC3 (AR−) prostate cancer cell lines, respectively. This level of activity is about 12, 70, and 40 times more powerful than that of cyproterone acetate, a known antiandrogen used as the control drug. Interestingly, the precursor **8a** was also active with an IC_50_ of 57 μM for LNCaP cells, 120 μM for DU-145 cells, and 132 μM for the PC3 cells. Dimers **6c**–**d** were less active than dimer **6a** but displayed selectivity on androgen-dependent LNCaP prostate cancer cells. Unfortunately, dimers **7a**–**c** were not tested in this study.

Comparative investigations of testosterone dimers **6b** and **7a** with similar chain lengths were recently performed in our laboratory [11,12,13,14,15]. The interactions with several bio-macromolecules were studied using various spectroscopic methods, transmission electron microscopy (TEM), as well as molecular modeling. The first study showed that beta-lactoglobulin was able to encapsulate testosterone readily in comparison with the dimers **6b** and **7a**. The binding affinity for beta-lactoglobulin was higher for testosterone with a binding constant of 5.6 × 10^4^ M^−1^ than **7a** with 2.9 × 10^4^ M^−1^ and **6b** with 4.8 × 10^3^ M^−1^ [11]. These dimers can also bind human serum albumin (HSA) and bovine serum albumin (BSA), proteins able to transport biological substrates and drugs [12,13]. The dimers could also alter DNA and tRNA morphology [14,15]. These investigations provide further insight into the nature of steroid–biological macromolecule interactions and demonstrate the value of designer dimers.

**Figure 4:**
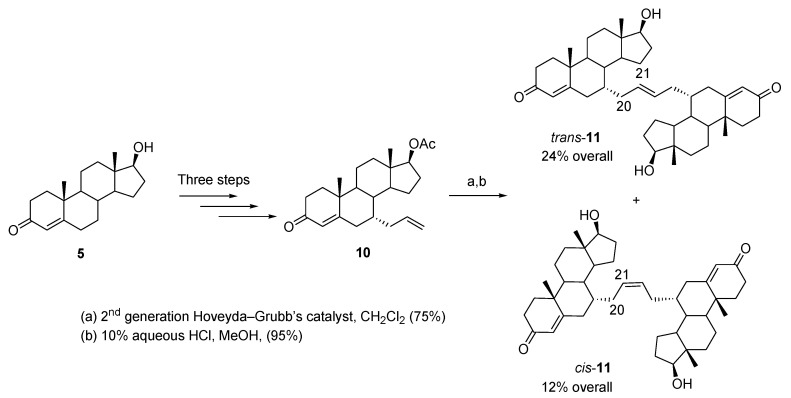
Synthesis of 7α-allyl testosterone acetate (**10**) and testosterone dimers *trans-***11** and *cis-***11** via an olefin metathesis reaction.

Bastien et al. reported the synthesis of two testosterone dimers [16]. They are readily available from testosterone (**5**) through an efficient five-step synthetic path with an overall yield of 36% (*trans-***11**, 24% and *cis-***11**, 12%) (Figure 4). The key dimerization step involved an olefin metathesis reaction of 7α-allyltestosterone acetate (**10**) with the Hoveyda–Grubbs second-generation catalyst. The isomeric dimers were easily separable by flash chromatography (*trans* and *cis,* 2:1). X-ray diffraction crystallography confirmed the structure of the minor *cis* isomer and indirectly proved the structure of the major *trans* isomer. MTT assays showed that the *cis* dimer had the best activity against human prostate cancer cell lines. *cis-***11** with an IC_50_ of 30.3 μM and 24.7 μM on LNCaP prostate cancer cells (AR+) and PC3 (AR−), respectively, displayed similar activity to that of the known antiandrogen cyproterone acetate. Interestingly, the *trans-***11** was active only on androgen-independent PC3 cancer cells.

In Denisov et al., the dimers were used to study allosteric effects in substrate binding to cytochrome P450 CYP3A4 by resonance Raman and UV-Vis spectroscopy [17]. This work shows that both dimers bind to the catalytic binding site of CYP3A4, which is known to be sufficiently flexible to accommodate structurally different substrates. It was discovered that the *cis*-**11** binds more tightly and induces about 100% spin shift due to its compact structure. In comparison, the *trans*-**11** is a larger molecule that binds similarly to two monomeric testosterone as it exhibits comparable spectral (resonance Raman (rR) spectroscopy) properties and binding affinity. This study provided the first direct evidence for an allosteric effect of the peripheral binding site at the protein–membrane interface on the functional properties of CPY3A4.

**Figure 5:**
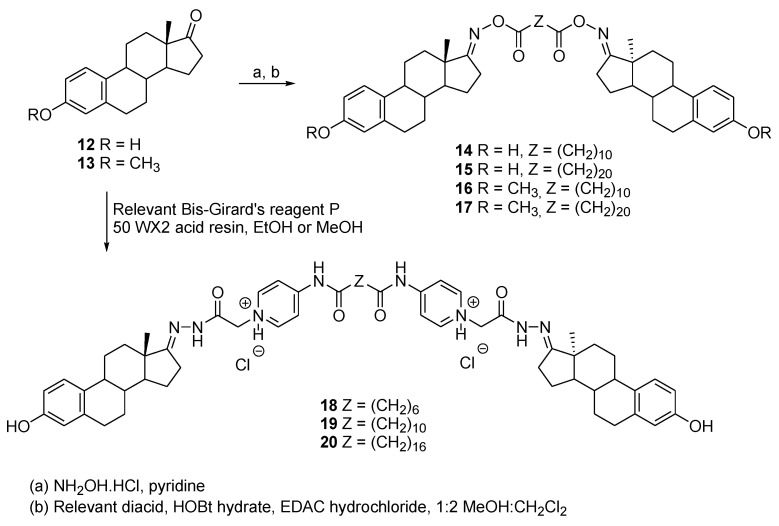
Synthesis of estrogen dimers (**18**–**20**) possessing amide linkers at the C-17 position.

In order to modulate estrogen receptor alpha (ERα)-mediated transcription events, Wendlant et al. developed a series of symmetric estrogen dimers linked at position C-17 [18]. The first series of dimers, comprised of compounds **14** to **17**, was fabricated through the use of oxime chemistry [19] by starting from estrone **12** or estrone 3-methyl ether **13** (Figure 5). A stability analysis was conducted and proved that the dimers were robust under various conditions. However, all compounds were analyzed for their agonist affinity for ERα and none of them showed a superior affinity to that of 17β-estradiol. In response to those results, dimers **18**, **19**, and **20** were synthesized in the hope of increasing ERα affinity. Those compounds were fabricated via the use of a Girard-based linker, which increases the hydrophilicity of the targets. Of all these compounds, the dimer **19** showed the best results with a binding efficacy to ERα of about 125% compared with that of estrogen at optimal concentration. This dimer was also evaluated for its binding specificity and was found to link strongly to ERα whereas it was inactive against the three steroid hormone receptors, which include progesterone receptors (PR), androgen receptors (AR), and glucocorticoid receptors (GR). In vivo studies are still needed to evaluate the drug potential of this new candidate.

**Figure 6:**
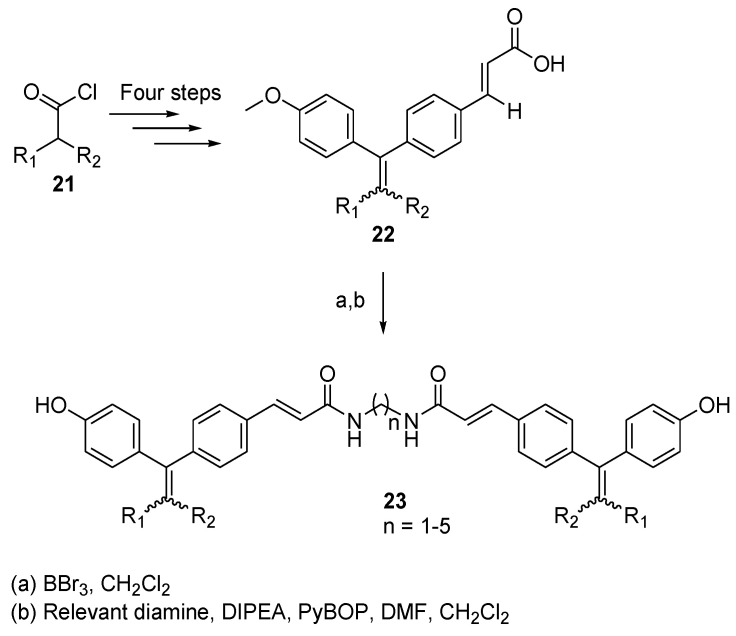
Synthesis pathway of the estrogen receptors antagonist (**23**) with corresponding starting materials (**21**).

With the aim of modulating the activity of the estrogen receptor (ER), a recent approach consisted of designing *C*_2_-symmetric dimers to bridge both ligand binding sites of a dimeric ER [20]. In order to exploit this strategy, Knox et al. developed a series of cyclophenylacrylic acid dimers, which can downregulate the activity of ER [21]. The choice of structure for those dimers was based on crystallographic and theoretical studies [22,23]. The synthetic route to form those dimers is shown in Figure 6. Starting from the relevant acyl chloride **21**, a Friedel–Crafts acylation with anisole followed by a Grignard reaction with 4-bromobenzaldehyde protected as acetal results in a compound that can be treated with acid to deprotect the acetal and dehydrate the hydroxyl group, which forms the diphenyl core. Then, a Wittig–Horner reaction with trimethyl-/triethylphosphonoacetate and hydrolysis allows the formation of compound **22**. The anisole ring is then converted to phenol and a treatment of the resulting compound with DIPEA, PyBOP, and the corresponding diamine spacer results in the formation of **23**. Of all the derivatives formed in the study, two principal series can be distinguished: the first corresponds to a derivative of GW7604 [23], where R_1_ is a phenyl group and R_2_ is an ethyl group; the second series is composed of cyclofenil derivatives [24], where R_1_ and R_2_ are linked by a cyclohexyl ring. In both these series, the number of carbons of the diamine spacer varies between one and five.

All compounds were tested in vitro for biological activity [21]. A transactivation assay showed that all compounds possess full antagonistic potency against ERα/β. The downregulative potential of the dimers was tested on the basis of ERα activity expressed in the MCF-7 cells and the results ranged from low to medium, every molecule being less potent than the reference drug fulvestrant, a known estrogen receptor antagonist. None of these compounds stimulated ER expression. The most active compound was the cyclofenil derivative that possesses a spacer containing four carbon atoms. Not only did this compound show the highest binding affinity to ERα with a relative binding affinity of 79.2% compared with fulvestrant, it also showed a downregulation efficacy of 38% at 1 µM against the ER content of the MCF-7 cells, as determined by an in-cell Western immunoassay. These results showed once again that the strategy of dimerization as an antagonist of a particular receptor may be an interesting method to synthesize novel medicinal compounds.

## 3. Sugars and Nucleoside-Based Dimers

**Figure 7:**
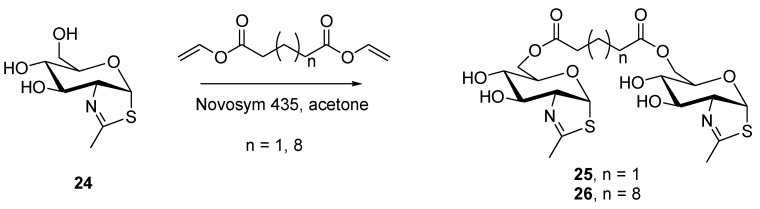
Enzymatic synthesis of NAG-thiazoline dimers **25** and **26**.

Sugars are essential to life, not only as a source of energy, but also as a building block for several bio-macromolecules. An interesting enzymatic synthesis of several C-6-acylated derivatives of NAG-thiazoline **24** (2′-methyl-α*D*-glucopyrano-[2,1-d]-Δ2′-thiazoline) was reported, and the products were tested for their inhibitory activities toward fungal β-*N*-acetylhexosamididase [25]. Amongst the products fabricated, two dimers were prepared by enzymatic transesterification of a suitable bis-vinylester and **24** using Novosym 435, a lipase from *Candida antarctica* immobilized on acrylic resin (Figure 7). The dimers **25** and **26** were obtained with 28% and 53% yield, respectively. The dimers were tested for their inhibitory activity on a fungal β-*N*-acetylhexosaminidase, and unlike the parent compound **24**, a well-known competitive inhibitor of the enzyme, both dimers displayed mixed inhibitory effects.

In a series of three recent publications, Barianiak et al. reported several nucleoside dimers analogues composed of floxuridine and thymidine linked by a 1,2,3-triazole ring system [26,27,28]. The target dimers are not symmetric but were designed as hybrid drugs with highly active antimetabolic building blocks. This research aimed at discovering compounds with enhanced biological activities. Particularly, the dimers could be used as nucleoside drugs, either by their chemical properties enabling them to terminate DNA synthesis or by their physical properties by disrupting the DNA helix. Overall, new anticancer drugs are sought by these researchers [26].

**Figure 8:**
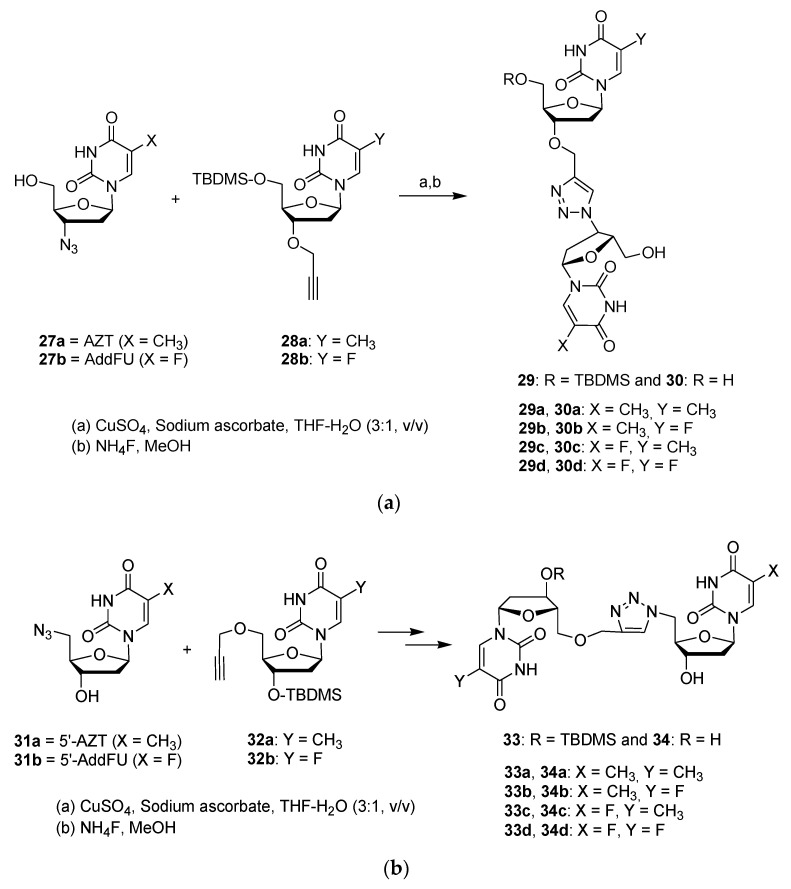
(**a**) Click chemistry synthesis of dimers **30a**–**d** by combination of 3′-azido-nucleosides and 3′-O-propargyl-nucleosides. AZT, 3′-azido-3′-deoxythymidine (**27a**); AddFU, 3′-azido-2′,3′-dideoxy-5-fluorouridine (**27b**). (**b**) Click chemistry synthesis of dimers **34a**–**d** by combination of 5′-azido-nucleosides and 5′-*O*-propargyl-nucleosides; 5′-AZT, 5′-azido-5′-deoxythymidine (**31a**); 5′-AddFU, 5′-azido-2′,5′-dideoxy-5-fluorouridine (**31b**).

Two types of dimers were formed: in the first type, the dinucleosides are linked at 3′–3′ position (**30a**–**d**); and the second type are connected at the 5′–5′ position (**34a**–**d**) (Figure 8a,b). Hence, the relevant azides (**27a**,**b** or **31a**,**b**) and propargyl ethers (either **28a**,**b** or **32a**,**b**) are reacted together using the Huisgen cycloaddition reaction to produce excellent yields (70–90%) for the dimers (**29a**–**d** and **33a**–**d**) bearing a 1,2,3-triazole ring system. Treatment with ammonium fluoride produce the final dimers **30a**–**d** and **34a**–**d** with 75–95% yields. The triazole ring replaces the natural internucleotide phosphodiester linkage, leading to greater stability by increasing resistance to nuclease enzymes. In comparison with the phosphodiester bond, the triazole ring is neutral, allowing increased cell penetration and interactions with DNA and RNA due to the lack of electrostatic repulsion [26].

The impact on cell viability of dimers and precursors was tested on three types of human cancer cells; KB (carcinoma nasopharynx), HeLa (cervical cancer), and MCF-7 (breast cancer) using the colorimetric MTT assay. The results were compared with the nucleoside drug cytarabine (ara-C), with an internal standard 5-fluoro-2′-deoxyuridine (5-FdU), and with 3′-azido-3′-deoxythymidine (AZT) as a control drug. It was discovered that dimer **34d** was the most active dimer with an IC_50_ of 3.10 μM on KB cells, 3.46 μM on MCF-7 cells, and 3.76 μM on HeLa cells. Dimer **34d** was twice as active as 5-FdU and displayed equipotent activity to that of ara-C. Dimer **34a** was also interesting with an IC_50_ of 3.40, 4.11, and 5.06 μM against HeLa, MCF-7, and KB cell lines, respectively. The 3′–3′ connection was less effective in producing active dimers than the 5′–5′ connection.

## 4. Dimers of Known and Synthetic Anticancer Agents

**Figure 9:**
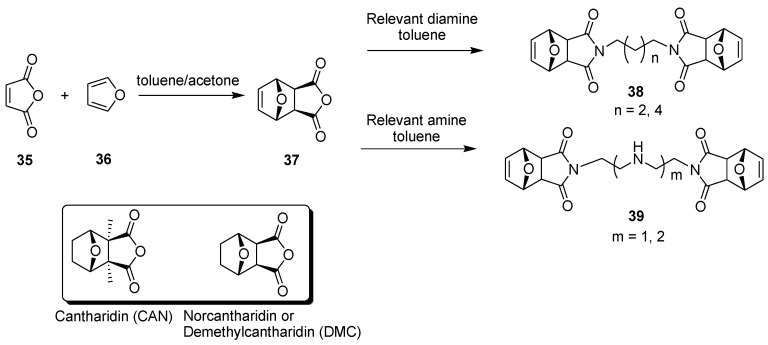
Synthesis of four unsaturated bis-norcantharimides: dimers **38** and **39**.

This section analyzes dimers formed with the goal of improving the anticancer effects of the monomeric unit. Simple small molecules such as cantharidin (CAN) and demethylcantharidin (DMC) are protein phosphatase inhibitors that have been used for centuries (since 1264) as anticancer agents against various cancer types [29], and the references cited there (Figure 9). These compounds are effective against multidrug-resistant cells; however, cantharidin is toxic to normal cells, primarily of the gastrointestinal tract, urethra, and kidney. So, many analogues were synthesized to improve its activity while reducing its toxic side effects on normal cells. Cheng et al. reported the synthesis and antiproliferative activity of four unsaturated bis-norcantharimides and the corresponding saturated molecules [29]. The synthesis is easy and consists of an initial Diels–Alder reaction between maleic anhydride (**35**) and furane (**36**) to give **37**, which is reacted with 1,4-diaminobutane, 1,6-diaminohexane, diethylenetriamine, and triethylenetetramine to obtain the final derivatives **38** (n = 2, 4) and **39** (n = 1, 2) (Figure 9). Catalytic hydrogenation provides the corresponding saturated dimeric analogues, but unfortunately, all these dimers were inactive on human lung cancer cells (A549) when tested by the cell viability MTT assay.

**Figure 10:**
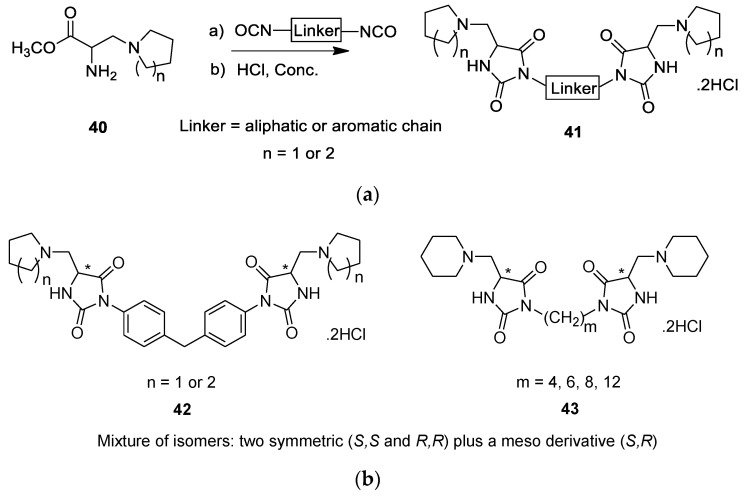
(**a**) General scheme for the synthesis of hydantoin dimers **41**. (**b**) Target dimeric molecules of symmetric hydantoin dimers **42** and **43**.

Furutachi et al. were interested in the design and biological evaluation of dimeric hydantoin dimers [30], and as seen in Figure 10a, the synthesis is straightforward [31,32]. These authors also reported previous work on symmetrical hydantoin derivatives [33]. In the most recent publication, the synthesis consists of reacting aminoester **40** with a relevant diisocyanide, providing a variety of hydantoin dimers **41** (Figure 10a) [30]. The target molecules are illustrated in Figure 10b. Two types of dimers were fabricated that are linked together either by an aromatic chain **42** or by an aliphatic chain **43** (Figure 10b), and the dimers were obtained as a mixture of stereoisomers. As a first screening, the authors tested the dimers on two types of human cancer cells: U251 (brain glioma cells) and KB3-1 human carcinoma cells [30], and if positive results are obtained, it is planned by the research group to separate the stereoisomers to more precisely study their biological potential.

The antiproliferative activity of the dimers was evaluated using the colorimetric MTT assay, which revealed that dimer **42** (n = 1) displayed the best activity with an IC_50_ of 0.46 and 5.21 μM on U251 and KB3-1 cell lines, respectively. This particular *C*_2_-symmetric dimer is connected by a biphenylmethane bridge. The IC_50_ of cisplatin, the reference drug, was 3.06 and 6.90 μM against these two cells, respectively. Generally, the hydantoin dimers were more active in U251 cancer cells with an IC_50_ ranging from 0.46 to 7.0 μM in comparison with an IC_50_ ranging from 5.21 to 26.08 μM on the KB3-1 cells. Notably, amongst the dimers linked by a methylene chain, dimer **43** (m = 8) showed the best antiproliferative activity on brain glioma cells (U251) with an IC_50_ of 1.05 μM. There was no clear relationship between the length of the aliphatic chain and the observed antiproliferative activities.

**Figure 11:**
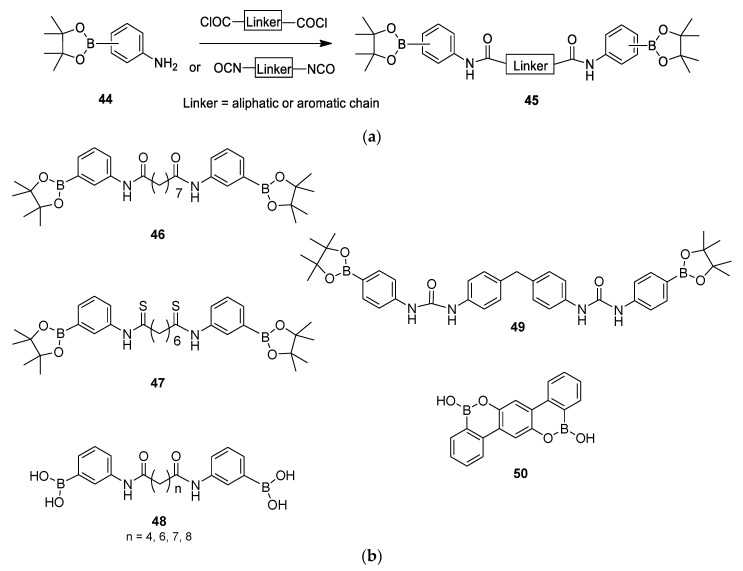
(**a**) Preparation of the phenyl boronic acid pinacol esters **45**. (**b**) Symmetric phenyl boronic acids (**48**, n = 4, 6, 7, 8), pinacol esters (**46**, **47**, **49**), and compound **50**.

A different research project from Furutachi et al. described the synthesis of *C*_2_-symmetric phenyl boronic acid pinacol esters with different linkers and reported their biological potential as antiviral and antibacterial agents [34], and more recently, as anticancer agents [35]. The general structure **45** is illustrated in Figure 11a. These dimers are easily prepared by reacting amino-phenyl boronic acid pinacol esters **44** with relevant dicarboxylic acid dichlorides in the presence of triethylamine (for example, leading to **46**) or with diisocyanide to obtain **49** (Figure 11b). Dimer **47** was synthesized by reacting the precursor bis-amide with Lawesson’s catalyst with 55% yield. A unique symmetric compound **50** was obtained with 35% yield from 2,5-diphenylhydroquinone reacted with BCl_3_ and AlCl_3_. The same research team also described several dimeric pinacol acids [36,37]. 

These molecules were tested for their anti-herpes simplex virus activities [34,36,37]. Interestingly, the phenyl boronic acid pinacol esters **46** showed anti-HSV-1 activity with an EC_50_ of 8 μM, while the corresponding phenyl boronic acid **48** (n = 7) was inactive (EC_50_ > 100 μM). The bis-thioamide **47** was twice as active as **46**, with an EC_50_ of 4 μM; also dimer **50** with an EC_50_ of 5.5 μM is an interesting anti-HSV-1 compound.

The new dimers were also tested for their anticancer activities on human brain glioma cells (U251) and human carcinoma cells (KB3-1) using the MTT assay [35]. The symmetric dimer **48** (n = 8) was the most active compound, displaying an IC_50_ of 19 and 3.78 μM on U251 and KB3-1 cancer cells, respectively. Of note, the antiproliferative activity of dimer **48** (n = 8) was greater than that of cisplatin (IC_50_ of 6.9 μM) on KB3-1 cells. Dimer **49** has a different linker chain and showed activity only on KB3-1 cells with an IC_50_ of 44.4 μM. Finally, dimer **48** (n = 7) showed moderate activity with an IC_50_ of 39.6 and 32.5 μM on U251 and KB3-1 cancer cells, respectively. There was no clear relationship between the length and nature of the linker with the observed antiproliferative activities. Overall, the synthesis of this type of dimer is easy and some of the compounds present interesting activity that could guide future development.

**Figure 12:**
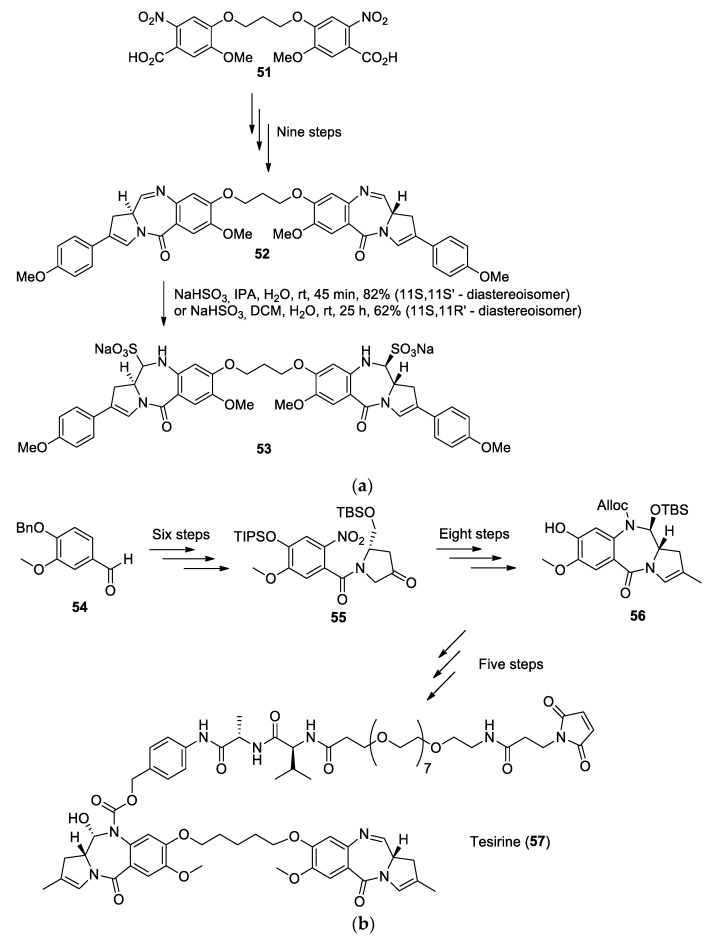
(**a**) SG2202 (**52**) and its prodrug SG2285 (**53**), two synthetic pyrrolo[2,1−c][1,4]benzodiazepine (PBD) dimers synthesized from the 2-nitrobenzoic acid dimer (**51**). (**b**) Scale-up synthesis of Tesirine (**57**) starting from benzylvanillin (**54**).

The antitumor properties of pyrrolo[2,1−c][1,4]benzodiazepine (PBD) compounds have been studied since 1963, when they were first isolated from the fermentation broth of the thermophilic actinomycete *Streptomyces refuineus* [38]. An initial biological screening of the extract showed a specific activity against Gram-positive organisms and an antitumor activity against sarcoma 180 and adenocarcinoma 755 mouse tumor systems [38]. It was later understood that the molecular structure of PBD compounds allows them to fit in DNA minor grooves and the electrophilic carbon of the imine group reacts with the amine of guanine bases, revealing the alkylating properties of PBDs [39]. In order to enhance their cross-linking properties, synthetic PBD dimers linked by their phenolic C8-positions via flexible ether bridge were investigated, since molecular modeling and NMR studies showed that this type of linkage could allow both PBD units to perform intrastrand or interstrand DNA cross-links, a hypothesis that was later reinforced by DNA-binding studies [39]. To that end, Howard et al. synthesized interesting PBD dimers in a nine-step synthesis (Figure 12a) by starting from the known 2-nitrobenzoic acid dimeric core (**51**) [40]. The key steps of this synthesis are the tetralactam formation, which was achieved with Raney nickel and hydrazine followed by a Suzuki coupling reaction. The resulting dimer SG2202 (**52**) was then tested in vitro, where it exhibited significantly higher cytotoxicity than other known PBD dimers [40]; however, the lack of hydrosolubility of SG2202 limited the in vivo assay. In order to resolve this problem, the prodrug SG2285 (**53**) was also synthesized by adding a bisulfite moiety at the C11 and C11′ positions. Both of these dimers were then tested on ten human tumor cell lines via an Alamar Blue assay. Although the prodrug SG2285 was slightly less effective than SG2202, both dimers showed a cytotoxic activity in the picomolar range for all cell lines tested, the best results being observed for the T lymphoblast cell lines CCRF-CEM, with an IC_50_ of 0.1 pM for SG2202 and 1.4 pM for SG2285 [40]. Further studies also demonstrated the cross-linking activity of SG2202 and SG2285 [41].

The biological potential of SG2285 (**53**) has sparked the attention of the scientific community in the last few years and its intellectual property has been acquired by Spirogen Ltd., London, UK. Following the outstanding results of SG2285, Spirogen developed other PBD dimers such as SG3249, also named Tesirine (**57**), a dimer first synthesized in 2012 [42] (Figure 12b).

Tesirine was designed to act as a warhead in the domain of antibody-drug conjugates (ADCs). In Tesirine, the PBD dimer acts as an antitumoral agent, whereas a valine–alanine linker is designed to be cleaved by Cathepsin B in order to release the chemotherapeutic drug in the body. Tesirine also has a polyethylene glycol (PEG) spacer and a maleimide designed to allow the conjugation of various antibodies via a Michael addition.

In 2016, Tiberghien et al. developed a scale-up synthetic route to Tesirine (Figure 12b) [43]. Key steps in their synthesis involve the nitration of benzylvanillin (**54**), followed by a Pinnick oxidation in order to form the carboxylic acid that can react with the hydroxyproline derivative to yield the corresponding amide. The hydroxyl group on the molecule is then oxidized with a TEMPO/TCCA combination, which results in the molecule **56**. Afterward, this compound is treated with triflic anhydride followed by a Suzuki coupling reaction to induce the methyl group at the C2 position. The nitro group was then reduced with zinc and dilute formic acid before being treated with allyl chloroformate to yield the corresponding carbamate (allyloxycarbonyl or alloc group). A deprotection of the hydroxyl group on the 2-pyrroline ring and a ring-closing Swern oxidation allowed the formation of the lactam, which was modified to form molecule **57**. This molecule was then subjected to Williamson ether chemistry via 1,5-diiodopentane in order to produce the PBD dimer. Prior to dimerization, one unit was linked with alloc-Val-Ala-*para*-amino-benzylalcohol. After the dimerization, the nitrogen bearing the alloc group was deprotected and the free amine was coupled with Mal-dPEG_8_-Acid to form Tesirine (**57**). With over 30 steps in total, it was possible for the authors to achieve this synthesis with a total yield of 0.54% [43].

The activity of Tesirine was studied and it was found that it exhibits cytotoxic effects in the ng/mL range against HER2 expressive human breast cancer cell line SKBR3 [43]. After more intensive biological studies [44], linkage of Tesirine with antibodies was tested and the antibody rovalpituzumab was chosen for its ability to bind to Delta-like ligand 3 (DLL3), an inhibitory Notch ligand expressed on the cellular surface of small-cell lung cancer and large-cell neuroendocrine tumors but expressed minimally in healthy tissues [45]. The drug candidate Rovalpituzumab Tesirine (Rova-T) was tested on small-cell lung cancer and demonstrated excellent cytotoxic activity [45]. Rova-T even progressed to clinical trials, but the developer AbbVie announced in 2019 that the Rova-T research and development program was ended in phase III clinical study due to a lack of survival benefit for the patients [46].

**Figure 13:**
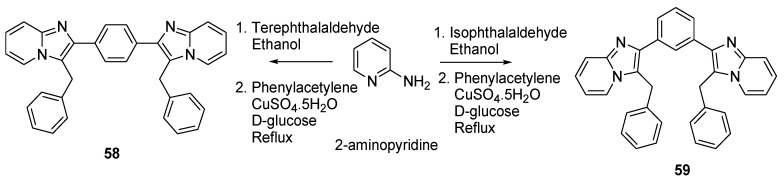
Reaction scheme of the transformation of 2-aminopyridine into the symmetric imidazo[1,2−*a*]pyridine dimers **58** and **59**.

Compounds containing Schiff base are known to often exhibit biological activity [47], and some drugs containing an imidazole motif that possess anticancer [48], hypnotic [49], and anxiolytic [50] properties are currently marketed. A series of Schiff-base dimers was developed in order to study the impact of dimerization on the biological activity of such compounds [51]. In this study, 30 dimers were synthesized and their effect was studied in three types of cancer cell lines. Of all the novel compounds, the dimers **58** (Figure 13) and **59** showed the best cytotoxic activity. These two compounds bear an imidazo[1,2−*a*]pyridine skeleton and were fabricated by a one-pot synthesis, where 2-aminopyridine reacts first with the corresponding dialdehyde to form the amidine, which is then heated in the presence of phenylacetylene, CuSO_4_, and *D*-glucose to yield the desirable compound [52].

Although many of the dimers appear to exhibit no significant cytotoxicity, compounds **58** and **59** showed high activity against the three cell lines tested: cervical (HeLa), breast (MDA-MB-231), and renal cancer cell lines (ACHN). In all cases, these dimers were found to possess an IC_50_ below 1 µM. In vivo studies were conducted with compounds **58** and **59** on mammary carcinoma rats and the parameters evaluated were hemoglobin, packed cell volume, red blood cells, standard deviation, and white blood cells. The results showed that these two compounds had similar activity to the tamoxifen reference, which highlights the therapeutic potential of symmetric imidazo[1,2−*a*]pyridine dimers.

## 5. Polyphenol Dimers

**Figure 14:**
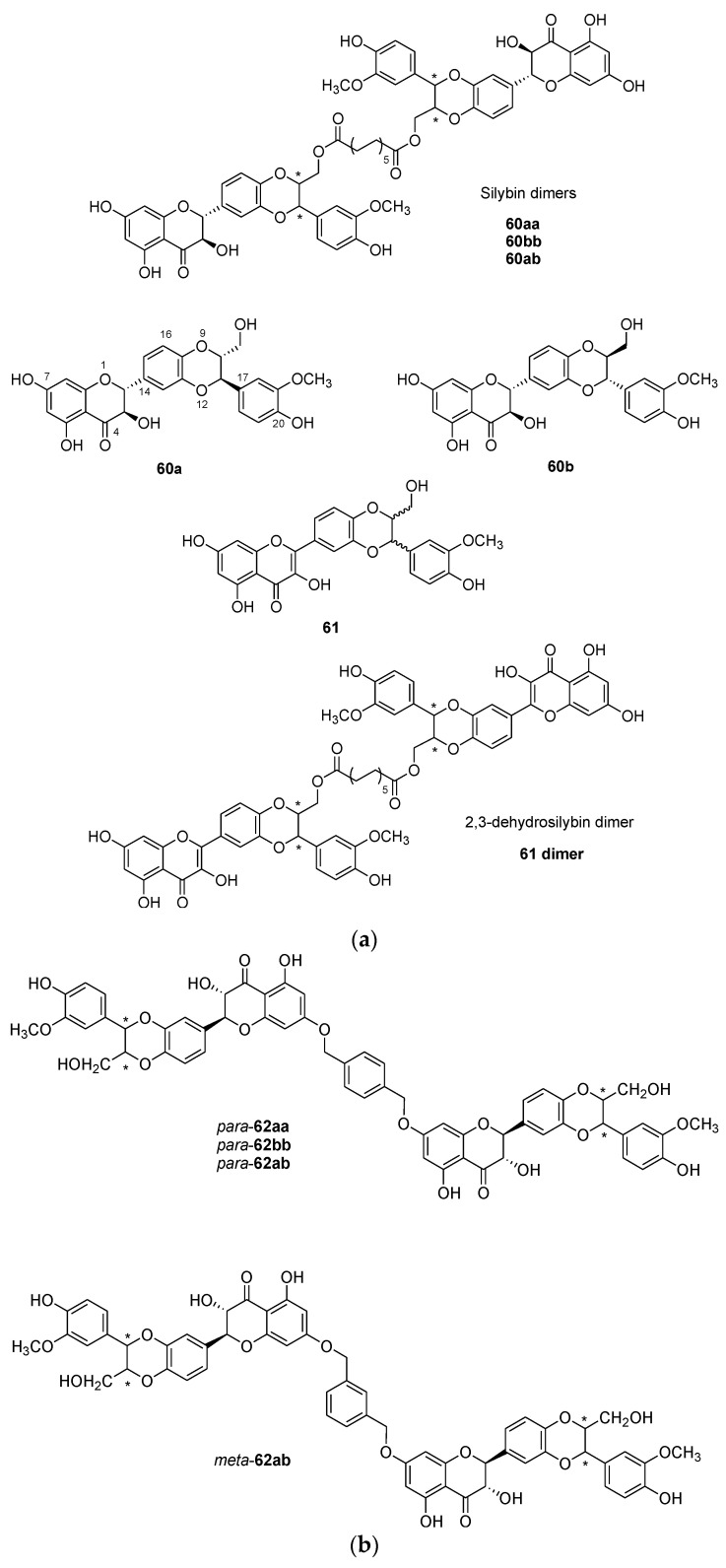

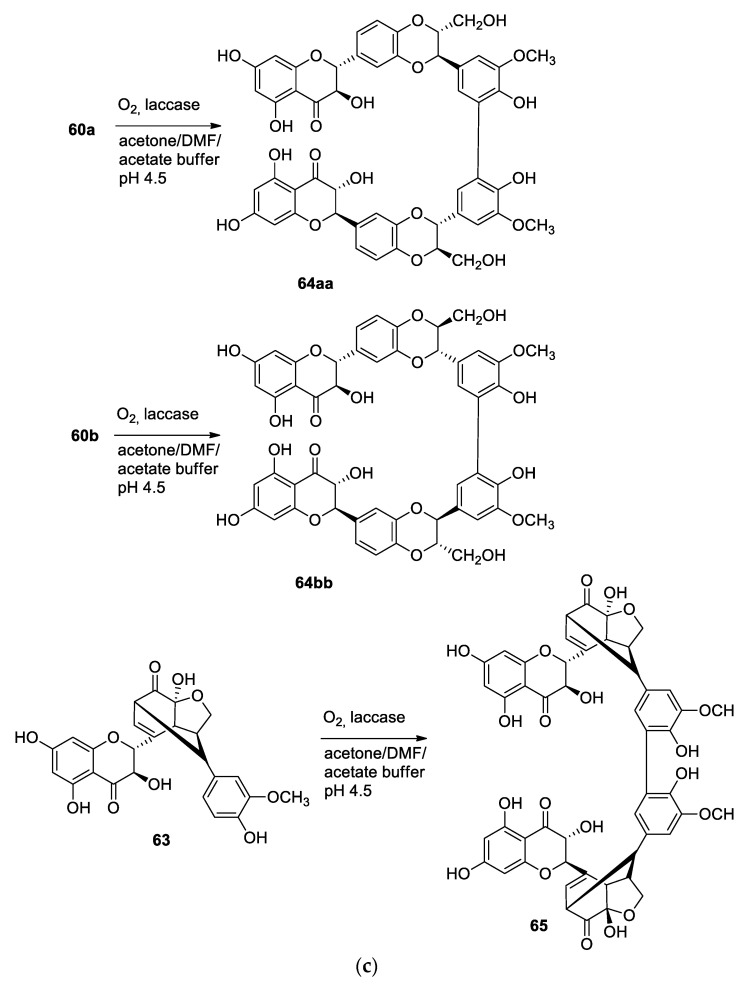

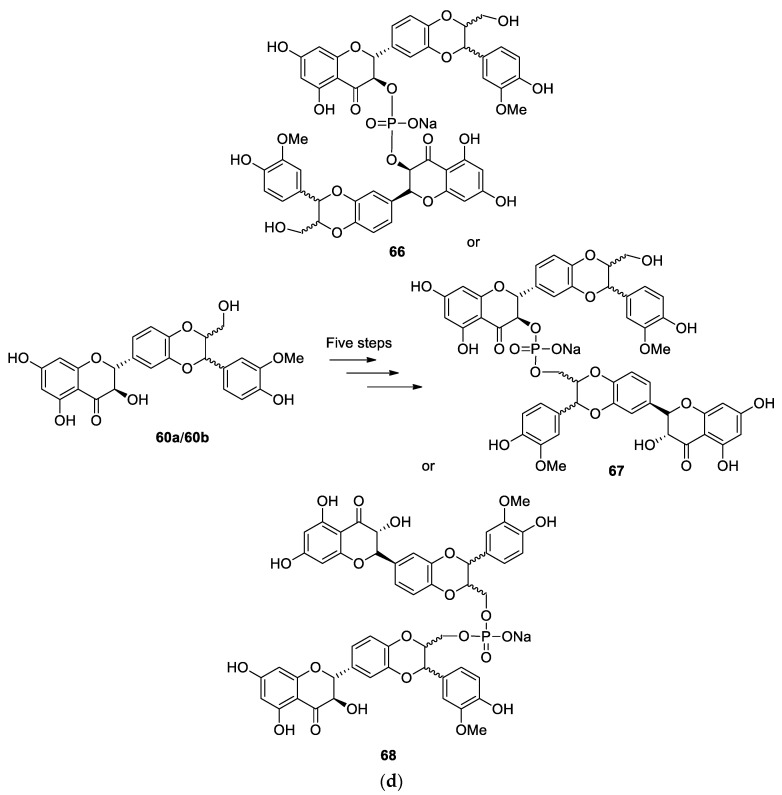
(**a**) Structure of silybin **60a** and **60b**, of 2,3-dehydrosilybin **61** and their dimers. (**b**) Silybin A and B dimers (**62**) with different combination linked at C-7 by a diether spacers. (**c**) Synthesis of symmetric flanovolignan dimers **64aa**, **64bb,** and **65** via oxidative coupling involving laccase from *Trametes versicolor*. (**d**) Synthesis of phosphate-linked silybin dimers **66**–**68** synthesized from silybin A/B **60a/60b**.

It is now established that flavonoids provide several health benefits, including anti-oxidative, anti-inflammatory, anti-mutagenic, and anti-carcinogenic properties, along with being able to modulate certain enzyme functions [53]. Silybin is a flavonolignan product extracted from the milk thistle (*Silybum marianum* (L.) Gaertn. (Asteraceae)), also named silymarin [54]. It is isolated as a mixture of two diastereoisomers silybin A (**60a**) and silybin B (**60b**) (Figure 14a), and they possess antioxidant and hepatoprotective activities. Interestingly, 2,3-dehydrosilybin **61** displays higher antioxidant and anticancer activities than silybin [55,56]. Generally, dimerization of these products (**60a**, **60b**) produces compounds with higher biological potential [54].

The *C*_2_-symmetric dimers **60aa** and **60bb** were prepared using a transesterification reaction with Novozym 435 and the divinyl ester of dodecanedioic acid in the presence of silybin **60a** or **60b** with 24% and 44% yields, respectively (Figure 14a). The novel dimers are linked at C-23 via a diester spacer and they are assembled by a lipase-mediated method [57]. Shorter divinyl ester did not provide any dimers but only monemeric esters. The asymmetric dimer was obtained in a stepwise manner via the monoester product of **60a**, which was then combined with **60b** to produce **60ab** in a 26% yield.

Another series of flavonolignans dimers was also developed that were linked by diether linkers either using 1,3-bis(bromomethyl)benzene or 1,4-bis(bromomethyl)benzene (Figure 14b). So, upon treatment of silybin A (**60a**) or silybin B (**60b**) with the relevant bis(bromomethyl)benzene and potassium carbonate in acetone at reflux, the dimers *para*-**62aa**, *para*-**62bb**, *para*-**62ab,** and *meta*-**62ab** were obtained as a diether linked at position C-7 (Figure 14b).

In order to evaluate antioxidant ability of the dimers, a 1,1-diphenyl-2-picrylhydrazyl (DPPH) assay was performed. The radical scavenging ability of the silybin dimers **60aa**, **60bb**, and **60ab,** and *para*-**62aa**, *para*-**62bb**, *para*-**62ab**, and *meta*-**62ab** (inhibition values varying between 7.1% and 10.7%) was superior to the inhibition value measured for silybin A (**60a**) (6.6%). However, the value determined for 2,3-dehydrosilybin **61** (83%) was much higher than the inhibition value of its corresponding dimer (see **61** dimer) (33.4%). The same trend in the results was observed in an inhibition of microsomal lipoperoxidation assay. The cytotoxic potential of silybin A **60a** and its dimer **60aa,** and 2,3-dehydrosilybin **61** and its dimer (**61** dimer) was tested on HUVEC vascular cells, NAK skin cells, BALB/c 3T3 fibroblasts, and HepG2 transformed hepatoma epithelial cells. Although all compounds were ineffective on the NAK cell line, silybin A (**60a**) was found to be less active than its dimer **60aa** on every other cell, and 2,3-dehydrosilybin **61** was more active than its dimer (**61** dimer). The authors rationalized the finding that dimerization of 2,3-dehydrosilybin **61** reduces its biological potential, whereas it enhances the potential of silybin A (**60a**), by the planarity of the flavonoid moiety of the 2,3-dehydrosilybin (**61**), which is much greater than that of silybin A. This planarity favors π-electron delocalization, leading to π-stacking within the dimeric molecule. Hence, the two flavonoid cores of the 2,3-dehydrosilybin dimer (**61** dimer) are much more prone to stacking than the monomeric units of dimer **60aa**, which can block some hydroxyl groups that are key components in the reactivity and antioxidant activity of these compounds, such as the resonance stabilization they can induce. Nonetheless, more assays are needed to grasp the importance of the stereochemistry of these flavonolignans dimers on their biological activity.

In another study, Gavezzotti et al. proceeded to dimerize the flanovolignans silybin A (**60a**), silybin B (**60b**), and silydianin (**63**) [58,59,60] at position C-21. In each case, the key step of the dimerization (Figure 14c) involves an enzymatic oxidative coupling using laccase from *Trametes versicolor*. The DPPH scavenging activity of the three dimers **64aa**, **64bb,** and **65** was tested, along with their corresponding precursors [60]. All the dimers show a better DPPH scavenging activity than their precursors, the most active compound being the dimer **65** with an IC_50_ of 7.92 ± 0.05 µM, a significant improvement compared with the substrate **63,** which showed an activity of 27.4 ± 0.7 µM. However, none of the activities of the compounds tested in this study surpassed the DPPH scavenging activity of the known antioxidant Trolox, which displays an IC_50_ of 4.18 ± 0.1 µM [61].

In order to enhance the solubility of silybin while retaining the properties of flavonolignans dimers, a series of three silybin dimers with phosphate linkers was developed [62]. The dimers were fabricated independently using a five-step reaction sequence involving phosphoramidite chemistry (Figure 14d) [63]. Three dimers, **66**, **67**, and **68**, were tested for antioxidant activity by DPPH tests. Every dimer was more active than the silybin **60a/60b** (IC_50_ of 1.40 ± 0.06 mM), the best one being dimer **68** with an IC_50_ of 0.34 ± 0.07 mM. However, all of the silybin derivatives were less active than the reference drug quercetin (IC_50_ of 0.18 ± 0.01 mM). Furthermore, the novel compounds were found to be non-cytotoxic against HepG2 cells and the solubility of the dimers was found to be around 20 mg/L at circumneutral pH values, which is a considerable improvement compared with silybin **60a/60b** that possesses a solubility around 0.4 mg/L. These results prove that polyphenol dimers are promising compounds in the field of synthetic antioxidants.

**Figure 15:**
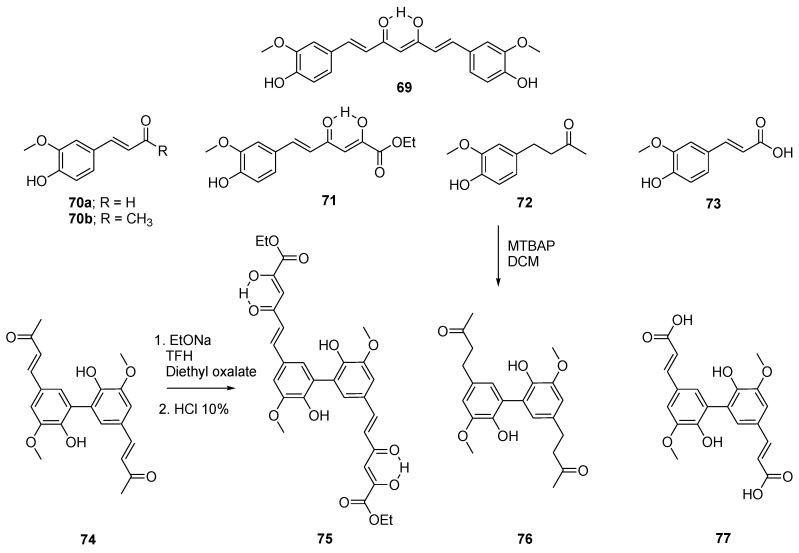
Representation of curcumin (**69**) and related compounds **70**–**77**.

Curcumin (**69**) has been proven to be one of the best antioxidants discovered in nature [64] but is known to decompose under physiological conditions [65]. Its degradation products mainly consist of vanillin (**70a**), dehydrozingerone (**70b**), and ferulic acid (**73**) (Figure 15) [65]. Following this discovery, a new research avenue was undertaken that involves the modification of these degradation products to create new antioxidants stable under physiological conditions [66]. These compounds are shown in Figure 15. Note that the dehydrozingerone dimer **74** was fabricated from dehydrodivanillin that was treated with an aqueous solution of LiOH in acetone [66], and that ferulic acid (**73**) and its dimer **77** were extracted from saponified maize bran and grass samples [67,68].

The curcumin derivatives were evaluated for their antioxidant properties [68]. The kinetic study of the autoxidation of triacylglycerols of sunflower oil showed that curcumin (**69**) and the *C*_2_-symmetric dimers **74** and **75** displayed stronger antioxidant efficiency and inhibition degrees than the other compounds and were more active than their corresponding monomers. However, an oxygen radical absorbance capacity assay with fluorescein was also performed, and with this model, dimers and monomers presented similar activity. Nonetheless, the monomers and dimers (**74**–**77**) showed superior activity to the reference Trolox at a similar concentration (0.63 µM). This assay was performed in water, which can greatly impact the results, since hydrogen bonding may affect the radical scavenging potential of the molecules. Structure–activity studies showed that the presence of an α,β-unsaturated chain seems to be a key component in the chain-breaking antioxidant activity of the compounds, since this moiety can scavenge the generated radicals. This effect also seems to be stronger when the α,β-unsaturated chain is located in *para*-position to a hydroxyl group on an aromatic ring. The authors concluded that since curcumin (**69**) and the compound **74** are two dimers originating from the monomer **70b**, the type of linkage that unites both phenolic units of these dimers does not change the reactivity or antioxidant efficiency of this type of molecule.

## 6. Terpenoid Dimers

**Figure 16:**
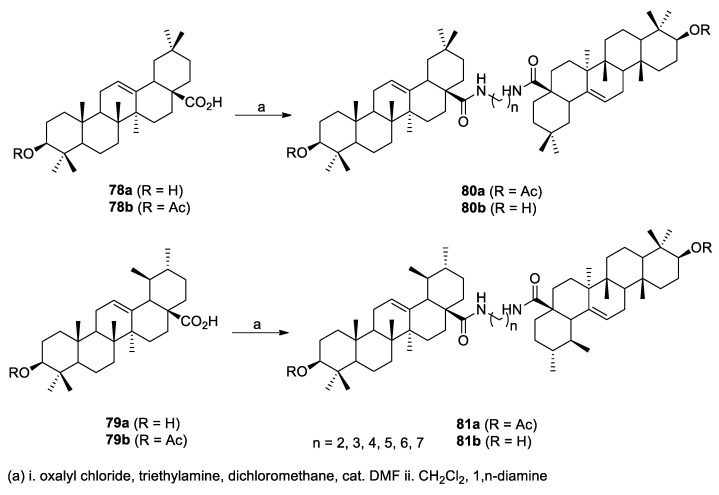
Ursolic acid (**78a**), oleanolic acid (**79a**), and dimers **80b** and **81b**.

Terpenoids are an important class of natural products with diverse biological properties (e.g., anti-inflammatory, antioxidant, and anticancer) and are often used in traditional herbal medicine. Several dimers of ursolic and oleanolic acid (**78a** and **79a**) were recently reported by Hoenke et al. [69]. They were formed with the goal of improving their anticancer potential. The symmetric dimers were linked by an α,ω-diaminoalkyl chain of various lengths leading to derivatives **80b** and **81b** (Figure 16). The acids were initially acetylated (**78b** and **79b**, R = Ac), linked with relevant diamine chain to produce **80a** and **81a** (R = Ac) and hydrolyzed to the final dimers **80b** and **81b** (R = H). Despite acelytated ursolic and oleanolic acid as well as some carboxamides showing better cytotoxic activity than the parent acids [70,71,72], none of the dimers were active when tested on human cancer cell lines (A375, HT29, SW1736, MCF-77, A2780, FaDu, and A549) and nonmalignant mouse fibroblasts (NIH 3T3). The authors are now investigating skin penetration, stability, and bioavailability of the dimers to be used as slow-release system for transdermal applications.

**Figure 17:**
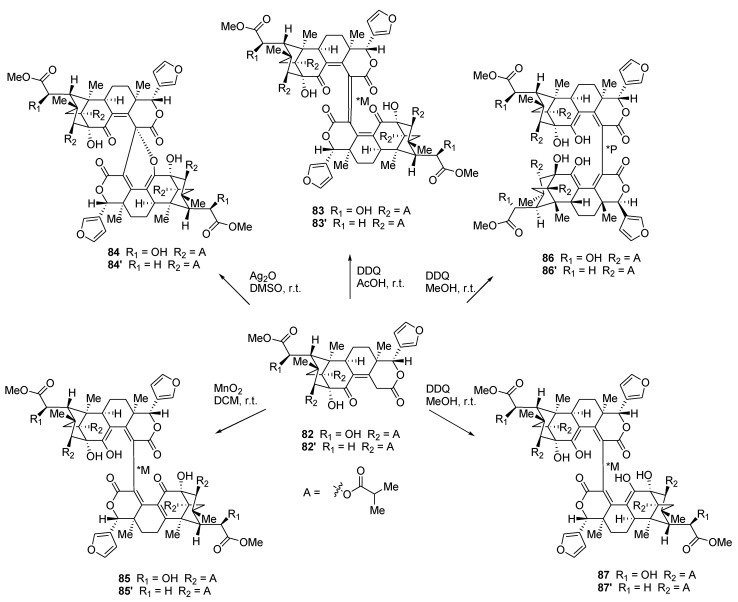
Formation of the limonoid dimers **83**, **83′**, **84**, **84′**, **85**, **85′**, **86**, **86′**, **87**, and **87′** along with their corresponding starting compounds 6*R*-hydroxymoluccensin A (**82**) and moluccensin A (**82′**).

Limonoids are categorized as highly oxidized tetranortriterpenoids, which mainly come from the plant families *Meliaceae*, *Rutaceae*, and *Cneoraceae* [73]. These compounds are also well-known for their important biological activity [74]. In 2017, Li et al. reported the discovery of the first limonoid dimer, named krishnadimer A (**87′**) (Figure 17), a *C*_2_-symmetric dimer linked by an axially chiral C15–C15′ central bond possessing an M-configuration. This compound was isolated from the mangrove *Xylocarpus moluccensis* [75]. Following this discovery, Li et al. synthesized a series of limonoid dimers, analogues of krishnadimer A (**87′**) [76]. Starting from moluccensin A (**82′**) [77] and 6*R*-hydroxymoluccensin A (**82**), the dimers **83**, **83′**, **84**, **84′**, **85**, **85′**, **86**, **86′**, and **87** were formed via oxidative carbon–carbon radical coupling. Krishnadimer A (**87′**) was also synthesized in order to obtain a sufficient quantity for biological assays.

Of all the dimers studied, only the compounds **83**, **83′**, **87**, and **87′** possess *C*_2_-symmetry. Every molecule was tested for cytotoxic activity against several human breast cancer cell lines. Compounds **84**, **84′**, **85**, **85′**, **86**, and **87** were found to be inactive on all cell lines and compounds **83** and **83′** were poorly soluble in DMSO and therefore could not be tested. Compound **82′** and **86′** were tested during a previous study and they showed no cytotoxic activity against eight human tumor cell lines [75]. Derivative **87′** was further tested on seven human breast cancer cell lines (MDA-MB-231, MDA-MB-453, MCF-7, MCF-7/ADR, MT-1, SK-BR-3, and ZR-75-1) [76]. Although it showed no activity on the last four cell lines, it displayed weak cytotoxic activity against MCF-7 cells (with an IC_50_ of 34.05 ± 7.35 μM) and showed potent activity against MDA-MB-231 (IC_50_ of 5.57 ± 1.48 μM) and MDA-MB- 453 cells (IC_50_ of 3.93 ± 0.75 μM), slightly exceeding the activity of cisplatin for this cell line (IC_50_ of 4.37 ± 0.32). The mechanism of action of **87′** was studied and it was discovered that this dimer induces cell-cycle arrest at G2/M phase and apoptosis. Moreover, an accumulation of reactive oxygen species (ROS) in MDA-MB-231 and MDA-MB-453 cell was measured. In vivo studies involving the transplantation of MDA-MB-453 cells into nude mice were conducted and **87′** successfully inhibited the growth of MDA-MB-453 tumors by 21.17% and 61.83% at doses of 10 and 30 mg/kg, respectively. It appears that *C*_2_ symmetry and the stable M-configuration of the C15–C15′ central axis are of utmost importance for the medicinal activity of **87′**.

## 7. Dimers of Known and Synthetic Antibacterial Agents

**Figure 18:**
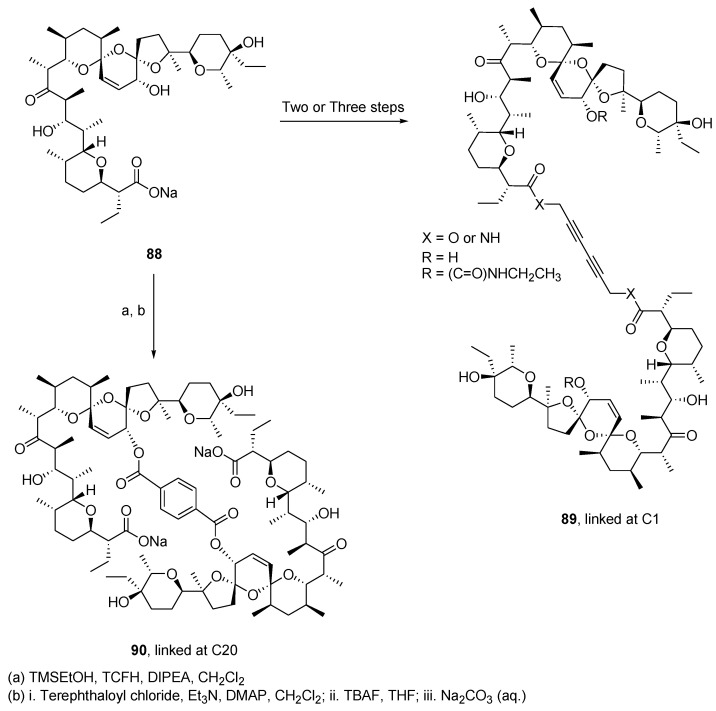
Synthesis of *C*_2_-symmetric salinomycin dimers **89** linked at C-1 and **90** linked at C-20 by a diyne chain and a terephthalate ester, respectively.

Salinomycin (**88**) is a polyether ionophore with a wide range of biological activities, primarily used in veterinary medicine as a coccidiostatic agent and growth promoter [78]. It was discovered that this natural product shows selective targeting of breast cancer stem cells [79] and anticancer potential against several human cancer cell lines [80]. These reports caught the interest of researchers and numerous semi-synthetic analogues were fabricated with the goal of obtaining compounds with better biological activity. Amongst this work, an early report of salinomycin dimers, linked by a triazole linker, displayed higher cytotoxicity against breast cancer cells than the corresponding monomer [81]. Hence, Antoszczak et al. prepared two different types of *C*_2_-symmetric salinomycin dimers by a rather expeditious and efficient method [82]. Four different salinomycin dimers were formed using a copper(I)-catalyzed Glaser-type reaction (general structure **89**) (Figure 18). These dimers were fabricated via the propargyl ester or amide of salinomycin with 83% and 88% yield, respectively, before the dimerization step using copper (I) chloride (CuCl). The C20 alcohol was either left intact or was protected as a carbamate using ethyl isocyanate as the reagent leading to two additional dimers. The C20-*O*-terephthalate dimer **90** was produced by a three-step reaction sequence with 36% overall yield. Of note, the Glaser reaction was achieved simultaneously in the presence of ethyl isocyanate used for the carbamoylation reaction.

The anti-proliferative activity of the dimers was evaluated using the SRB assay on human colon carcinoma (LoVo, doxorubicin-sensitive LoVo/DX, and doxorubicin-resistant) on three breast cancer cell lines (JIMT-1, MCF-7, and SKBR-3) and on the normal-like breast epithelial cell line (MCF-10A). Unfortunately, the four dimers (see **89**) were essentially inactive. However, dimer **90** displayed activity similar to that of salinomycin (**88**) and was more active than cisplatin. To illustrate its activity, the IC_50_ reported for MCF-7 breast cancer cells are: salinomycin, 1.5 μM; **90**, 1.8 μM; cisplatin, 7.7 μM; and doxorubicin, 0.26 μM. Interestingly, the dimer **90**, with an IC_50_ of 21 μM, was much less toxic than doxorubicin with an IC_50_ of 0.58 μM on normal-like breast epithelial cell line MCF-10A. Furthermore, **90** displays an IC_50_ of 2.8 μM in comparison with 10 μM for doxorubicin on LoVo/DX.

**Figure 19:**
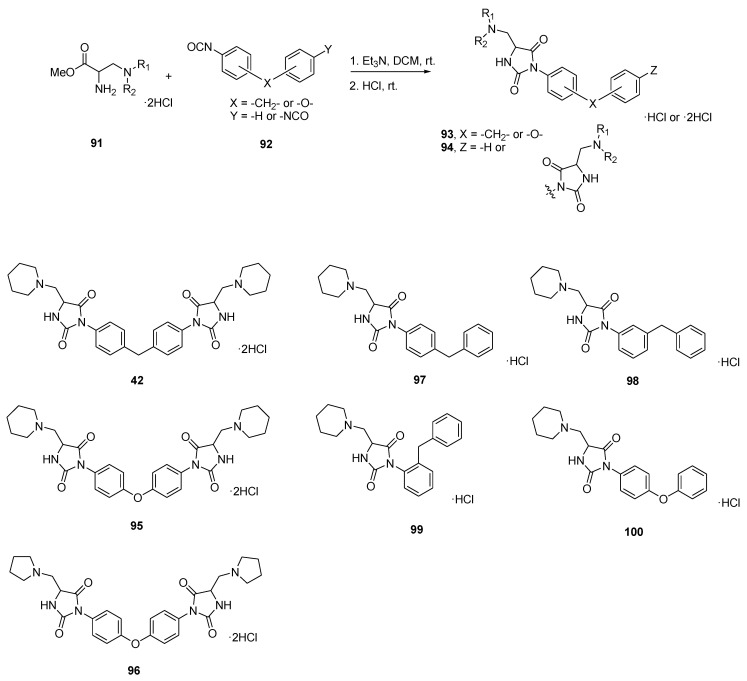
Reaction scheme of the synthesis of the hydantoin motif and representation of the various hydantoin derivatives.

In the aim of enhancing the antibacterial properties of hydantoin type drugs [83], Furutachi et al. synthesized a series of seven hydantoin derivatives (**42**, **95–100**), with three of these compounds possessing a *C*_2_-symmetry axis (**42**, **95**, **96**) [84] (Figure 19). The compounds were all produced by the reaction of a β-aminoalanine derivative **91** with various aryl isocyanates **92** leading to general structures **93** and **94** (Figure 19) [31]. The new compounds were tested on a Gram-positive (*S. aureus*) and a Gram-negative (*E. coli*) strain for antibacterial activity. Of all these compounds, the three most potent candidates are the *C*_2_-symmetric dimers (**42**, **95**, and **96**), the best one being compound **42**, which exhibited a minimum inhibitory concentration of 24 nM against *S. aureus* and 95 nM against *E. coli*. Furthermore, every compound tested was more active against the Gram-positive strain *S. aureus* than the Gram-negative *E. coli*, for reasons that remain to be elucidated.

**Figure 20:**
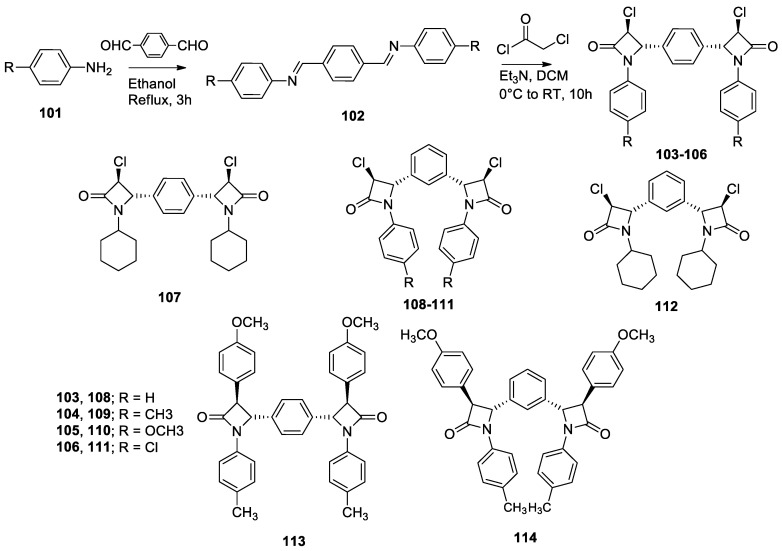
General scheme of the synthesis of *C*_2_-symmetric *β*-lactam dimers and representation of novel compounds **103** to **114**.

Compounds containing the β-lactam ring, such as penicillin or cephalosporin, are known to possess antibacterial activity due to their capacity to inhibit the synthesis of bacterial cell walls [85]. More recently, these compounds were also proven to possess certain antitumor activity [86]. A series of symmetric β-lactam dimers were produced by reaction of an aromatic dialdehyde with various amines **101** to yield a series of Schiff bases **102** (Figure 20) [87]. These imines were then treated with chloroacetyl chloride, or the corresponding acyl chloride, in order to form compounds **103** to **114**, illustrated in Figure 20. Those novel molecules were exposed to nine different bacterial strains to examine their antibacterial activities. The results varied from low to medium, the most active dimer being molecule **109**, which notably showed an inhibition zone of 26 mm for the strains *Proteus vulgaris*, *Salmonella typhi*, and *Staphylococcus aureus*. However, some intermediate compounds involved in the formation of dimers **113** and **114** were more active than the dimers, and none of the novel compounds were more active than the reference drugs amoxicillin and ceftriaxone, which had an inhibition zone of 30 mm in every tested strain. Tested for their in vitro anticancer activity, the compounds showed variable results. The best results was obtained with dimer **104** with IC_50_ values of 0.41, 0.42, and 0.45 µM against cervical HeLa, breast MDA-MB-231, and renal ACHN cell lines, respectively. These results are even higher than that obtained for doxorubicin, which highlights the biological potential of β-lactam dimers in the discovery of new anticancer drugs.

## 8. Recently Isolated Dimeric Natural Products

**Figure 21:**
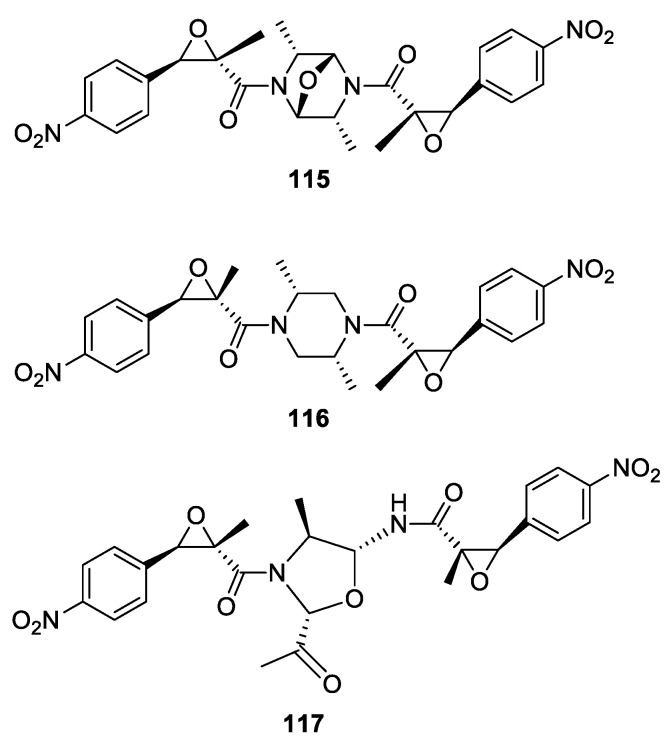
Natural products with two nitrophenyl *trans*-epoxyamides, chrysamides A−C (**115**–**117**) isolated from the deep-sea fungus *Penicillium chrysogenum*.

Three natural products possessing two *trans*-epoxyamide were isolated from the deep-sea fungus *penicillium chrysogenum* (**115**–**117**) [88]. The structures of the compounds were characterized by IR, ^1^H-, and ^13^C-NMR spectroscopy and by mass spectrometry. X-ray diffraction analysis of compounds **115** and **116** was performed, confirming the spectral analysis. Interestingly, two of the dimers (**115** and **116**) feature a center of symmetry (Figure 21).

The compounds were tested for their anticancer activity against K562, A549, and HUH7 cancer cell lines and were inactive at 30 μM. They were also tested for their antibacterial activity against three bacteria (*Staphylococcus aureus*, *Escherichia coli*, and *Salmonella* sp.) and were inactive. Interestingly, compound **117** showed anti-inflammatory activity, inhibiting the production of pro-inflammatory cytokine IL-17, with an inhibitory rate of 40% at 1 μM. Compounds **115** and **116** did not display any inhibitory effects at 50 μM.

**Figure 22:**
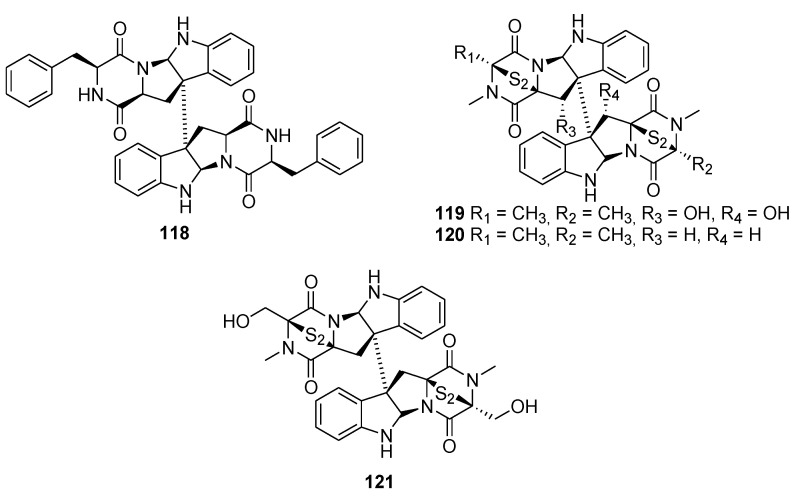
Representation of biologically active diketopiperazine dimers produced by marine microorganisms.

Although the medicinal properties of diketopiperazine produced by marine microorganisms have been extensively studied in the past few years [89,90], the biological and biochemical interest in diketopiperazine dimers (Figure 22) produced by those same microorganisms is lacking. One of such compounds is WIN 64821 (**118**), a molecule that can be extracted from *Aspergillus sp*. [91]. Even though WIN 64821 presents a certain cytotoxic activity on 37 human cancer cell lines [92], the biological interest in this molecule is because it exhibits the capacity to compete with Substance P (SP) to act as an antagonist of NK-1 receptor with an IC_50_ of 0.24 µM [93]. Derivatives of WIN 64821 were also synthesized by Barrow et al. before being tested for binding activity with the NK-1 receptor, and none of them showed affinity for the receptor nearly as potent as WIN 64821, which shows that the symmetry of this molecule is a key component in its biological activity [93].

Another pair of interesting dimers are Verticillin A (**119**) and 11,11′-dideoxyverticillin A (**120**). Both of these compounds are found in *Penicillium* sp., a marine-derived fungus [94]. Both of these dimers exhibit a diketopiperazine moiety along with an interesting disulfide bridge inside the piperazine skeleton. Although both of these dimers share a similar structure, they do not possess the same biological activity. Verticillin A (**119**) demonstrates an interesting anticancer activity against pancreatic ductal adenocarcinoma (PDAC) and colon carcinoma due to its ability to inhibit the following histone methyltransferases (HMTases): SUV39H1, SUV39H2, G9a, GLP, NSD2, and MLL1 [95,96]. Furthermore, in vitro and in vivo assays demonstrated that Verticillin A has the capacity to suppress metastatic colon carcinoma that displays chemoresistance to 5-fluorouracil [95]. The same study showed that Verticillin A also has the potential to overcome colon carcinoma that expresses resistance to FasL-induced apoptosis and can increase death receptor 5 (DR5), which leads to an effective suppression of resistance to DR5 agonist drozitumab-induced apoptosis [95]. The cell-free ELISA tyrosine kinase assay demonstrated that 11,11′-dideoxyverticillin A (**120**) has the capacity to inhibit the activity of vascular endothelial growth factor receptor-1 (VEGFR-1) and epidermal growth factor receptor (EGFR) with an IC_50_ of 1.645 ± 0.885 nM and 0.136 ± 0.109 nM, respectively [97]. Those results demonstrate that 11,11′-dideoxyverticillin A has potent antitumor activity.

Another molecule of interest is chaetocin (**121**). Although its structure is similar to those of Verticillin A and 11,11′-dideoxyverticillin A, chaetocin is from marine-derived fungus *Nectria inventa* [98]. A unique property of this mycotoxin is its ability to act as a competitive inhibitor of *S*-adenosylmethionine by inhibiting SU(VAR)3–9 with an IC_50_ of 0.6 µM [99]. Chaetocin also has the ability to inhibit SUV39H1, which has led some research teams to combine chaetocin with other epigenetic drugs to develop new therapeutic strategies against certain types of cancer, notably leukemia [100]. Moreover, it was also proved that chaetocin may induce cellular oxidative stress, mainly by inhibiting the redox enzyme thioredoxin reductase. An NCI-60 screening demonstrated that chaetocin can effectively inhibit cellular proliferation in solid tumor along with inducing apoptosis in every solid tumor tested by an oxidative damage mechanism [101].

**Figure 23:**
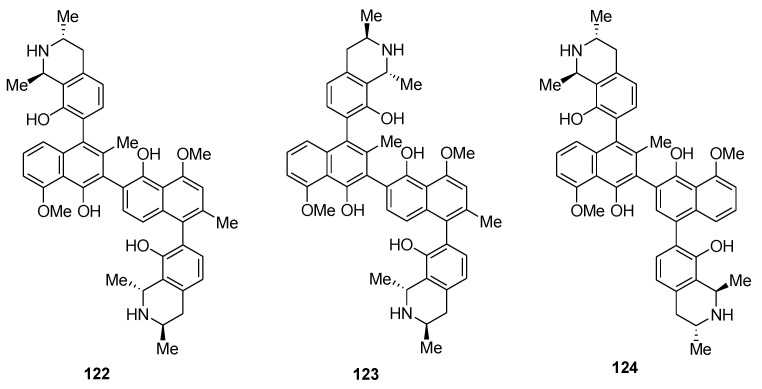
Representation of the naphthylisoquinoline dimers jozilebomines A (**122**), jozilebomines B (**123**), and jozimine A_2_ (**124**).

The medicinal properties of naphthylisoquinoline alkaloid compounds extracted from Central African plants have already been well-studied. [102]. Li et al. reported the discovery of novel naphthylisoquinoline dimers extracted from the roots of the Congolese plant *Ancistrocladus ileboensis* (Figure 23) [103]. These dimers are jozilebomines A (**122**) and jozilebomines B (**123**). They were extracted along with the already known dimer jozimine A_2_ (**124**), a *C*_2_-symmetric dimer that was isolated in 2013 from a Congolese *Ancistrodadus* species, which was the only known dioncophyllaceous dimer discovered in nature prior to the discovery of jozilebomines A and B [104]. The elucidation of the structure of jozilebomines A and B was achieved by 1D and 2D NMR, HRESIMS, oxidative degradation, and ECD data. All three dimers were tested for their cytotoxic activity against HeLa human cervical cancer cell line. The most potent compound is jozimine A_2_ (**124**, IC_50_ of 0.22 μM), followed by jozilebomines B (**123**, IC_50_, 0.68 μM) and jozilebomines A (**122**, IC_50_, 1.08 μM). The dimers were also tested for their activity against the PANC-1 human pancreatic cancer cell line and again, jozimine A_2_ (**124**) was found to be the most effective compound (IC_50_ of 0.10 μM), better than jozilebomines B (**123**, IC_50_, 0.87 μM) and jozilebomines A (**122**, IC_50_, 2.24 μM). In this case, the activity of jozimine A_2_ was even stronger than that of the reference drug arctigenin (IC_50_ of 0.83 μM). Finally, the antiplasmodial activity of the dimers was studied on a small series of protozoan parasites and all the dimers displayed some antiplasmodial activity. The most active molecules were Jozilebomines A and Jozilebomines B with an IC_50_ of 0.043 μM and 0.102 μM, respectively. However, these results are less promising than the antiplasmodial activity of jozimine A_2_ (IC_50_ of 1.4 nM), which was evaluated on the strain NF54 of *Plasmodium falciparum*. These results demonstrate that *C*_2_ symmetry is a key component in the biological activity of these compounds. With jozimine A_2_ (**124**) being the naphthylisoquinoline alkaloids with the best antiplasmodial activity, its mechanism of action is currently under investigation [105].

## 9. Summary and Conclusions

This review presented recent advances in the design of *C*_2_-symmetric biologically active molecules, which is a topic currently attracting the attention of many scientists. The main goal of designing dimeric drugs is the discovery of a compound with enhanced biological activity. A dimer may induce strong biological activity such as the silybin A dimer **60aa**, which was more active than the parent molecule **60a** on all cells tested, with the exception of skin cells (NAK) [54]. In addition, the testosterone dimer **6a** tested on three prostate cancer cell lines displayed activity 12 to 70 times stronger than that of the reference drug, cyproterone acetate [10]. Another interesting example is the pyrrolo[2,1−c][1,4]benzodiazepine dimers **52** and **53**, which displayed cytotoxic activity in the picomolar range for all cancer cell lines tested [41]. Nevertheless, in some cases, dimerization leads to poor activity, such as for the unsaturated bis-norcantharimides dimers **38** and **39** [29] and for the ursolic and oleanolic acid dimers **80b** and **81b** [69]. The main cause for low biological activity is generally due to low solubility and bioavailability of the resulting compounds; hence, dimers of highly soluble drugs should be first considered as strong candidates for the likely outcome of potent new medicine. In this regard, natural products are a good source of hydrophilic biologically active molecules. Notably, isolation of marine natural products has led to the discovery of unique dimeric compounds such as the nitrophenyl *trans*-epoxyamides and the diketopiperazine [88,93]. Dimers extracted from plants can also lead to promising compounds, as is the case for jozimine A_2_ (**124**) [104,105].

Another important consideration for designing dimeric drugs should be ease of synthesis. This can be achieved by classic reactions such the formation of ether bonds by Sn2/Sn1 reactions, ester or amide formation, anhydride chemistry, isocyanate chemistry, click chemistry, oxidative coupling of aromatic ring systems, etc. There is no doubt that a dimeric drug can produce higher biological activity, but the synthetic path should be short, efficient, and readily translated to the pharmaceutical industry. Otherwise, as we saw in this review, the goal of discovering a potent drug might simply not be attained. In summary, future developments in this field must be based on (i) careful selection of monomers, (ii) knowledge of mechanisms of action, and (iii) efficient synthesis leading to the desired dimeric molecules.

## Data Availability

Data available in a publicly accessible repository.
